# Classification of Sonar Targets in Air: A Neural Network Approach

**DOI:** 10.3390/s19051176

**Published:** 2019-03-07

**Authors:** Patrick K. Kroh, Ralph Simon, Stefan J. Rupitsch

**Affiliations:** 1 Chair of Sensor Technology, Friedrich-Alexander-Universität Erlangen-Nürnberg, 91052 Erlangen, Germany; stefan.rupitsch@fau.de; 2 Department of Ecological Science, Vrije Universiteit Amsterdam, 1081 HV Amsterdam, The Netherlands; ralph.simon@vu.nl

**Keywords:** sonar measurements, sonar detection, neural networks, feature extraction

## Abstract

Ultrasonic sonar sensors are commonly used for contactless distance measurements in application areas such as automotive and mobile robotics. They can also be exploited to identify and classify sound-reflecting objects (targets), which may then be used as landmarks for navigation. In the presented work, sonar targets of different geometric shapes and sizes are classified with custom-engineered features. Artificial neural networks (ANNs) with multiple hidden layers are applied as classifiers and different features are tested as well as compared. We concentrate on features that are related to target strength estimates derived from pulse-compressed echoes. In doing so, one is able to distinguish different target geometries with a high rate of success and to perform tests with ANNs regarding their capabilities for size discrimination of targets with the same geometric shape. A comparison of achievable classifier performance with wideband and narrowband chirp excitation signals was conducted as well. The research indicates that our engineered features and excitation signals are suitable for the target classification task.

## 1. Introduction

Air-based ultrasonic sonar sensors are often deployed for obstacle avoidance and navigation purposes in application areas such as automotive, factory automation as well as mobile ground and airborne robotics [1,2,3]. In these application areas, ultrasonic sonar sensors show distinct advantages in comparison to other sensor technologies, which are based on other physical principles, such as LIDAR (Light Detection And Ranging), cameras and RADAR (Radio Detection And Ranging) sensors. Most importantly, they are not susceptible to obstacles’ optical and electromagnetic properties. Therefore, they do not depend on obstacle color, lighting as well as transparency or material-related radar cross section. Another important aspect is that direct physical contact is not necessary. Consequently, ultrasonic sensors are especially well-suited for robot operation in low-visibility scenarios, e.g., in a dark room and outdoors at night, and for avoidance of transparent plastic or glass obstacles. Ultrasonic sensors may be included as primary sensors or as vital components in a complementary sensor fusion setup that comprises additional sensors. Such a setup would combine the different sensors’ advantages, which may thus lead to improved system robustness.

Often, ultrasonic sensors are only applied to determine the distance to closest obstacles [4] due to their low angular resolution in comparison to other sensors, but by incorporation of distinct acoustic targets, we aim to facilitate landmark-based localization as well as mapping and make position predictions more precise [5,6]. Incorporation of fixed landmarks is particularly beneficial for navigation in unstructured and changing environments. This may be the case for living assistance robots at home (ambient assisted living [7,8]), where the environment is constantly changed by people. Another use case can be indoor as well as outdoor robot farming applications, in which vegetation changes its shape due to plant growth and farming activities, such as cutting off branches and harvesting fruits [9,10,11].

In order to realize landmark-based navigation, it is first necessary to perform target identification, on which this article puts its main emphasis. For this, geometrically different shaped as well as sized targets are deployed and classification is performed on these. We insonify the targets with narrowband as well as broadband chirp signals, emitted by an electrostatic speaker, record the echoes with a measurement microphone, classify the echo signals and compare the classification results. Broadband signals are used since their cross-correlation functions are narrow and they are in general better suited to deduce spectral object features than narrowband signals due to larger frequency ranges that are covered in a single echo. Likewise, echolocating bats are also known to emit broadband signals as soon as they have to resolve objects in front of vegetation [12]. It was shown that they are able to classify different geometrical objects independent of their size [13,14] as well as same geometrical objects of different sizes [15].

Artificial neural networks (ANNs) are employed as classifiers. ANNs represent an interesting option as they are able to generalize from a limited amount of training data and are known to cope well with noisy data. Additionally, ANNs are also able to learn features from raw data that are not obvious to a human observer and which might thus remain unaccounted for in a merely rule-based classifier. We engineered features based on target strength (TS) and also incorporated preprocessed raw data input for our feature vectors, so that feature learning is possible as well [16,17,18].

Notable research in this area was conducted by Ayrulu et al. who performed tests with ANNs for obstacle detection with single-frequency pulses from piezoelectric sensors. They focused on engineered features, which were based on echo time of flight as well as magnitude and showed possible merit of amplitude features [19,20]. Dmitrieva et al. performed sonar target classification with chirp signals in water. They classified spherical targets that consist of different materials and demonstrated that ANNs perform best for their application in comparison to other machine learning-based classifiers, such as support vector machines [21]. Eliakim et al. also used broadband chirps for robot navigation. They utilized generic features from audio processing for binary obstacle classification (“plant/no plant” [22]).

In this contribution, we present specific recognition of multiple different targets in air with a comparison of narrowband as well as broadband chirps with ANNs as classifiers, based on specifically engineered TS features as ANN input. In the referenced literature, only one type of excitation signal is used in each article (single-frequency/narrowband [19,20] or wideband [21,22]) and no comparison is attempted. In addition, generic features for speech recognition [22], alternative raw-data representations [19,20,21] and engineered amplitude features [19,20] are fed into ANNs but no directly calculated target strength estimate features, which are based on pulse-compressed echo signals in our case. First results from the authors are published in [23]. The aim of our work is to engineer suitable features, perform target classification and compare feature quality based on classification results, so that suitable features for acoustic landmark identification can be selected as well as optimized.

## 2. Materials and Methods

Echo measurements were performed for different target positions and angles. During these, a signal was emitted by an ultrasonic speaker, reflected off a target, then received by a measurement microphone and digitized by an ADC interface card. Calculations for feature preprocessing and target classification were conducted on a desktop computer with Matlab R2018b.

### 2.1. Measurement Setup and Procedure

**Figure 1:**
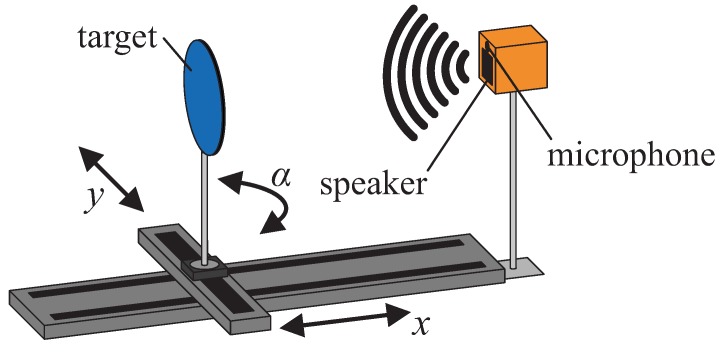
Measurement setup (not true to scale), with translation (*x*, *y*) and rotation (α) stages.

The measurement setup (as shown in Figure 1) consists of a two-axis translation stage, a rotation stage on top of the translation stage on which the targets were attached, a 1/4” Bruel&Kjaer measurement microphone (Type 4939-A-011) with an amplifier (G.R.A.S. 12AK), a wideband electrostatic ultrasonic speaker (Senscomp 7000 series, also formerly known as capacitive *Polaroid* transducers [24]) with a custom-built high voltage amplifier (0 V–400 V, 0 kHz–200 kHz, sine) and a National Instruments data acquisition device for analog IO (NI-USB-6356, 1.25 MS/s, 16 bit). The speaker is capable of sound emission in a frequency range of 20 kHz up to more than 150 kHz at sound pressure levels above 85 (with the standard reference value of 20 μPa for sound in air), which has been experimentally verified.

As an alternative, transducers based on ferroelectric materials, such as PVDF (Polyvinylidene Fluoride) [25,26,27] and EMFi (Electro Mechanical Film) [28,29], may be used as they are also suitable for wideband ultrasound emission, but must be custom-built. The custom-built amplifier is necessary as the ultrasonic speaker requires a bias voltage of 200 V and a maximum peak–peak voltage of 400 V. The microphone and the speaker are mounted closely together (20 mm center distance) at the end of the *x*-axis translation stage. All measurements were performed in an anechoic chamber, so there was no influence of other sound sources from outside the chamber. It has to be noted that the chamber walls are optimized for absorption of audible sound but strongly reflect ultrasound waves. As a consequence, our whole measurement setup had to be optimized especially so that there is no detectable direct echo from itself nor the walls that would interfere with the main target echoes. This includes that the targets, microphone and speaker are located 1 m above the floor. Moreover, all parts are placed with the largest distance possible to the closest walls (at least 1 m). Surfaces facing the setup are covered with Basotect material, which absorbs acoustic waves in the ultrasonic range. In doing so, it could be achieved that echoes resulting from multiple reflections, appear after the target echoes in the measured waveforms and do not interfere with these.

**Figure 2:**
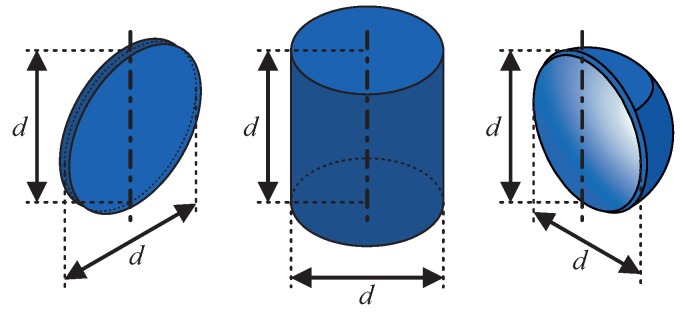
Target shapes (disc, cylinder and hollow hemisphere) with characteristic dimension *d* and rotation axes (0${}^{{}^{\circ}}$ for objects facing speaker/microphone with "flat" side, arbitrary for cylinder due to rotation symmetry).

For ANN training, validation and test, sample echoes were required. The targets were automatically moved along a grid and were also rotated—*x*-direction (0.5 m to 1.8 m, 0.1 m steps); *y*-direction (−0.15 m to 0.15 m, 0.05 m steps); angles (α, −60${}^{{}^{\circ}}$ to 60${}^{{}^{\circ}}$, 15${}^{{}^{\circ}}$ steps, compare Figure 2). We applied downward modulated, rectified, wideband chirp signals for electrical excitation of the electrostatic Senscomp speaker (*wb*, 150 kHz to 20 kHz, 1 ms duration). In additional measurements, narrowband chirp signals (*nb*, 52 kHz to 48 kHz, 1 ms duration) were employed since we are also interested in the performance that may be achieved if a common narrowband ultrasonic sensor is utilized, such as a piezoelectric-based transducer [30]. Chirp signals were chosen as they make it possible to gain information regarding a large portion of the spectrum from a single echo.

### 2.2. Targets

**Figure 3:**
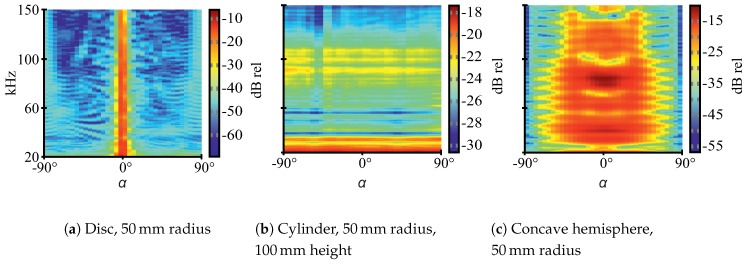
Echo fingerprints (spectral target strength vs. angle) for different target shapes at a distance of 1 m.

We collected and characterized ultrasound echoes from six different targets. Those can be grouped into three basic target shapes (Figure 2): flat, convex and concave. The mentioned grouping was chosen because the shapes show quite different reflective behavior with respect to insonification angle and echo magnitude, as visualized by the acoustic fingerprints in Figure 3. The targets in [20] can be grouped accordingly. More details regarding the acoustic fingerprints will be examined later in this section. As we wanted to use basic and generic shapes, we chose to use discs, cylinders and hollow hemispheres. For each shape, we analyzed two different sizes so that binary classification was performed as far as size discrimination is concerned. The characteristic dimension *d* was chosen to be 60 mm and 100 mm, respectively. The disc thickness is 4 mm and the cylinders as well as the hemispheres have 2 mm wall thickness. All targets were manufactured with a 3D printer (Ultimaker 2) and consist of ABS plastic.

The three different geometric shapes show characteristic acoustic fingerprints (Figure 3)—spectral TS versus rotation angle plots (see Section 2.4 for detailed explanations regarding TS). At flat targets, a single reflection occurs which shows low angular spread and high magnitude. At convex targets, there is also a single reflection but with wide angular spread and, consequently, lower magnitudes for the reflected wave as its energy is distributed across a larger volume. Inside concave targets, multiple reflections are possible which may lead to specific spectral properties due to interference [15]. Concave targets can also have retroreflecting properties, such as a corner reflector for radar systems. As a consequence, echoes for the selected shapes should significantly differ in magnitude and spectral composition, particularly with respect to the insonification angle. Thus, the shapes should be well-distinguishable. More detailed explanations regarding the hollow hemispheres’ acoustic properties can be found in [15].

Note that the depicted acoustic fingerprints are for illustration purposes only and are based on additional measurements, whose data is not part of the ANN training data. The measurement procedure for the acoustic fingerprints is different as well. The data for the fingerprints was obtained at 1 m distance between speaker and targets (“echoes”) or speaker and microphone (“transmission”), respectively. The targets were rotated from −90${}^{{}^{\circ}}$ to 90${}^{{}^{\circ}}$ at 5${}^{{}^{\circ}}$ steps (compare Figure 2 for 0${}^{{}^{\circ}}$ orientation) and swept single-frequency sine burst excitation was used with a frequency step size of 0.5 kHz. To obtain TS values, the ratios between transmission and echo RMS values are calculated. This approach is necessary for reliable results since the employed electrostatic ultrasonic speaker shows noticeable harmonic distortion at all excitation frequencies, which has been observed in laboratory measurements. Thus, before RMS calculations for the echo fingerprints, the signals are narrowly bandpass-filtered at each center frequency so that only the relevant baseband components are considered.

### 2.3. Neural Networks

**Figure 4:**
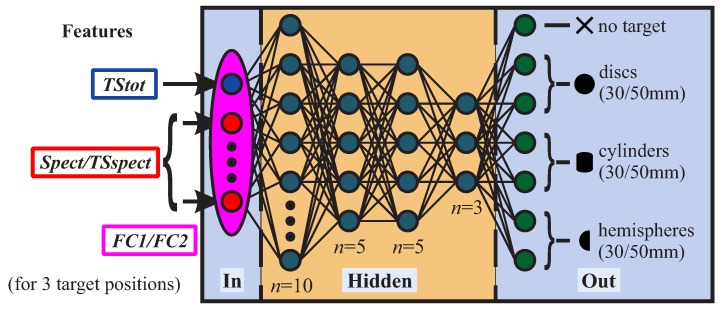
ANN architecture, with features, input, hidden as well as output layers, output classes and node numbers *n*.

Multi-layer perceptrons (MLPs) are applied as ANNs (Figure 4). We selected MLPs on purpose since they are more susceptible to variations in feature quality in comparison to other types of ANNs and should hence be better suited for estimates of feature performance. Our ANNs comprise four hidden layers in order to achieve good generalization and to avoid overfitting to training data. Due to the multi-layer structure, more meaningful and thus compressed information for the classification task needs to be represented/passed by each node if compared to a single layer network with the same total amount of nodes. This reduces the risk of overfitting to the training data [16,17]. The hidden layers comprise 10, 5, 5 and 3 neurons, respectively. To obtain a well-working network, we utilized a randomized parameter study in which several different hidden layer as well as node numbers were tested. The selected network architecture shows the best performance for all tested feature sets. Risk of overfitting was also minimized by choosing a low number of nodes for the hidden layers in comparison to other networks that are applied for similar classification tasks [21,22].

Seven output classes were created—one for each target and a separate one for non-target samples. For each class, roughly the same number of samples was created so that there is an even distribution among the classes. A scaled conjugate gradient backpropagation algorithm with a crossentropy error function was chosen for training (see [16,17] and [18] for details). Supervised learning is performed as the target positions as well as the target classes are known from the measurement procedure (see Section 2.1). Therefore, labeled data sets could be created. Of each dataset, 20 are picked for training, 10 for validation and 70 for testing. We chose the mentioned distribution as a larger training set might result in overfitting due to redundant echoes caused by symmetry in the measurement setup.

ANN performance evaluation is based on prediction accuracy, precision, recall as well as $F_{1}$ scores for test sets that have no common samples with their corresponding training or validation sets (see [16,17] and [18] for details). The measures can be obtained from confusion matrices, which show classification result counts, grouped by actual input classes and predicted output classes. Accuracy is to be maximized towards 100 and is defined as (compare [16,31,32] and [33]).

(1) $$\begin{array}{c} a=\frac{n_{\mathrm{hit}}}{n_{S}}\;\;\;, \end{array}$$

where *a* denotes the total accuracy, $n_{\mathrm{hit}}$ the total number of correct classifications in the test set and $n_{S}$ the total number of samples in the test set. Accuracy alone is usually not sufficient as a performance measure because it is only a measure for overall performance but does not contain information on ANNs’ performances for different classes. Thus, an ANN’s overall performance can seem very good, but it will still be possible for single classes to be identified very badly if multiple classes exist and especially if data set sizes vary for different classes. Hence, for evaluation of single-class classification performance, precision and recall are applied. Precision and recall values are both to be maximized towards 100% and are calculated for each target class by the given equations (compare [16,31,32] and [33]).

(2) $$\begin{array}{cc} p & =\frac{n_{\mathrm{TP},\mathrm{cls}}}{n_{\mathrm{FP},\mathrm{cls}}+n_{\mathrm{TP},\mathrm{cls}}} \end{array}$$

(3) $$\begin{array}{cc} \mathrm{and}\;\;\;r & =\frac{n_{\mathrm{TP},\mathrm{cls}}}{n_{\mathrm{FN},\mathrm{cls}}+n_{\mathrm{TP},\mathrm{cls}}}\;\;\;, \end{array}$$

in which *p* is precision, *r* is recall, $n_{\mathrm{TP},\mathrm{cls}}$ is the total number of true positives for a given class, $n_{\mathrm{FP},\mathrm{cls}}$ is the total number of false positives for a given class, and $n_{\mathrm{FN},\mathrm{cls}}$ is the total number of false negatives for a given class. Precision is a measure for certainty of correct classification for a sample of a specific output class, whereas recall is the percentage of correctly identified samples of available samples for a target class. Recall is also known as sensitivity in statistics [33]. The meaning of the terms true positive, true negative, false positive and false negative shall be illustrated. For the multi-class case here, we always consider the current target class, for which precision and recall are calculated, as positives and all other classes are summarized as negatives. This means for the mentioned terms:

- true positive: correct classification of current target class (e.g., hemisphere classified as hemisphere),
- true negative: correct classification of current non-target class (e.g., non-hemisphere classifed as non-hemisphere, where non-hemisphere may be anything but a hemisphere),
- false positive: wrong classification of current non-target class (e.g., non-hemisphere classifed as hemisphere),
- false negative: wrong classification of current target class (e.g., hemisphere classifed as non-hemisphere).

Evaluation of feature performance based on precision and recall can be cumbersome since a 2D plot must be created for each trained ANN. As a consequence, the $F_{1}$ score is introduced as a scalar measure, which combines precision and recall. The $F_{1}$ score is to be maximized towards 100 and is calculated as

(4) $$\begin{array}{c} F_{1}=2\;\frac{p\;r}{p+r}\;\;\;. \end{array}$$

Care must be taken for classes with small sample counts, but in our case this does not apply as our samples are evenly distributed among the different classes. For more detailed explanations regarding ANNs and their performance measures, see [16,17] and [18].

### 2.4. Echo Preprocessing and Features

**Figure 5:**
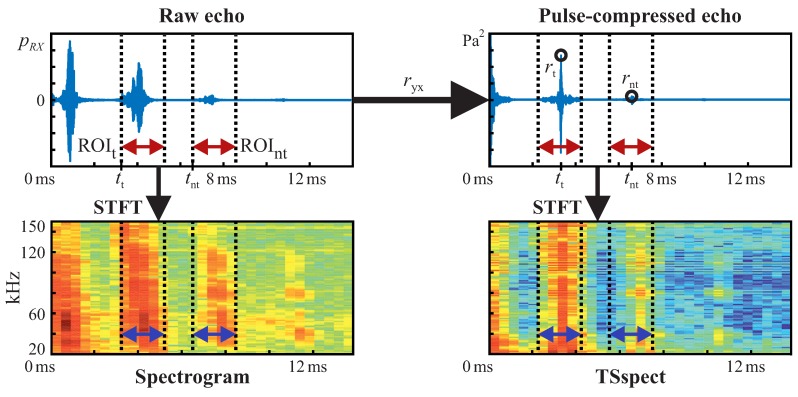
Main calculation steps from raw target/non-target echo pressure signal $p_{\mathrm{RX}}$ to spectrogram feature (*Spect*), total TS estimate feature (*TStot* via $r_{t}$/$r_{\mathrm{nt}}$ from cross-correlation $r_{\mathrm{yx}}$ with excitation signal) and spectral TS estimate feature (*TSspect*). ROIs (${\mathrm{ROI}}_{t}$/${\mathrm{ROI}}_{\mathrm{nt}}$) are illustrated by dotted lines and enclosed arrows. Echo delays are indicated by $t_{t}$ and $t_{\mathrm{nt}}$.

The recorded raw echo signals (top left in Figure 5) are processed before classification by ANNs. The signals are preprocessed since known relations can be extracted from data efficiently with rule-based approaches and so the ANNs do not need to learn those relations from the training data. Accordingly, learning focuses on aspects of information that are not modeled explicitly but may be important for the given classification task. The data which contains relevant information is part of the input to ANNs and is generally denoted as “input feature vector” or just “features” in the machine learning context [16,17,18]. Possible input features are raw data, alternative raw data representations (e.g., based on transformation to frequency or time-frequency spaces) as well as raw data on which basic mathematical operations are performed, such as multiplication of elements, squaring elements, calculation of various norms etc. Such features are often used for deep learning [16]. In addition, there are also specifically engineered features that are motivated by domain knowledge (common for traditional machine learning and pattern recognition [17,18]) as well as combinations of previously mentioned features. For our application, we created and evaluated aforementioned feature types, which are explained later on in this section. The main echo preprocessing and feature calculation steps are sketched in Figure 5. First, bandpass filtering is performed according to the excitation signal bandwidth. This is done to remove out-of-band noise, which is not related to the target echoes and is not relevant for classification. Then, each echo signal’s cross-correlation $r_{\mathrm{yx}}$ with the corresponding emitted acoustic chirp signal is calculated (top right part of Figure 5). Therefore, much irrelevant/unrelated in-band information is filtered out as well, such as noise and uncorrelated sounds from other sources. Consequently, the ANNs do not need to learn how to filter out a large portion of the information that is not relevant. The peaks in correlated signals can now be considered echoes from targets. Accordingly, peak positions correspond to targets’ propagation delays. This approach is also known as pulse compression in the area of sonar and radar systems (see also [34,35,36], and [37]). The emitted chirp signal had been recorded before at 1 m distance and was averaged ten times to improve its signal to noise ratio—in contrast to the measured target echoes, for which no averaging was performed (single echoes).

From each of the recorded echoes (raw as well as pulse-compressed), a region of interest (ROI) of 2 ms is selected for feature calculations (indicated by dotted lines with enclosed arrows in Figure 5). For each target’s echo, its ROI (${\mathrm{ROI}}_{t}$ in Figure 5) is centered around the largest corresponding peak in its pulse-compressed echo. Peak detection in pulse-compressed echo signals is a common method for identification of possible targets in sonar as well as radar systems (see also [34,35,36] and [37]) and is therefore assumed to be a valid step before target classification in our case. ROIs for non-target samples (${\mathrm{ROI}}_{\mathrm{nt}}$ in Figure 5) are randomly put outside target ROIs, but inside possible ranges for target echoes. Non-target ROIs are also centered around their highest peak in the pulse-compressed waveforms. ROIs in general are selected so that feature calculations do not need to be performed on the whole recorded waveforms, which would lead to larger ANN input feature vectors and therewith increased calculation cost. Another advantage of ROI selection is that only parts of the recorded signals are presented to the ANNs, which are actually related to the targets. So, no features are learned which may result from the measurement setup, such as additional echoes that might appear due to multiple reflections off targets and the setup. In doing so, training only happens on relevant parts of our echo signals and the risk of problems due to long feature vectors is largely reduced, e.g., overfitting due to curse of dimensionality [16,17,18]. The ROI length does not represent the proposed sonar’s working range, which was tested up to a target distance of 1.8 m in our case. Therefore, total echo recording durations were set to 15 ms. The distance limitation is given due to the sizes of the translation stages as well as the anechoic chamber dimensions (compare also Section 2.1). Tests are planned for greater distances outside the anechoic chamber in the future.

In particular, we consider three features:

(i) ***Spect***: the raw echoes’ spectrogram representation (bottom left plot in Figure 5),
(ii) ***TStot***: an estimate of total TS and
(iii) ***TSspect***: a specifically engineered feature, which is related to the targets’ spectral TS (bottom right plot in Figure 5).

*TStot* is based on the pulse-compressed echoes’ peak magnitudes ($r_{t}$, $r_{\mathrm{nt}}$ in Figure 5), whereas *TSspect* is based on a short-time fourier transform (STFT) of the pulse-compressed echoes. Specific relations will be shown later in this section and in the Supplementary Materials in detail. For *Spect* and *TSspect*, a frequency ROI was selected according to the excitation chirps’ bandwidths. Based on the mentioned features, we defined different feature sets for performance comparison:

- ***TStot***: *TStot* only,
- ***Spect***: *Spect* only,
- ***FC1***: combination of *Spect* and *TStot*,
- ***TSspect***: *TSspect* only,
- ***FC2***: combination of *TSspect* and *TStot*.

A feature vector was chosen to consist of a feature set for three adjacent measurement grid positions. This is equivalent to a sonar sensor traveling by a target and recording multiple echoes (e.g., robot passing by). Also, to each feature vector, the echo propagation delay is added as additional element. In order to obtain 1D-Feature vectors for our ANNs, 2D data is flattened into 1D arrays.

We chose the spectrogram (STFT) as time–frequency-based raw data representation since this is a common approach for speech recognition and because ANNs are also known to handle pictorial information quite well [38,39,40,41,42]. Another possible time-frequency signal representation is the wavelet transform, which was examined by Ayrulu and Barshan in [20]. *TStot* and *TSspect* are related to an object’s TS, which is a relative measure for acoustic intensity reflected off a target. A compact description of the relations is given in the rest of this section, more detailed explanations can be found in the Supplementary Materials.

**Figure 6:**
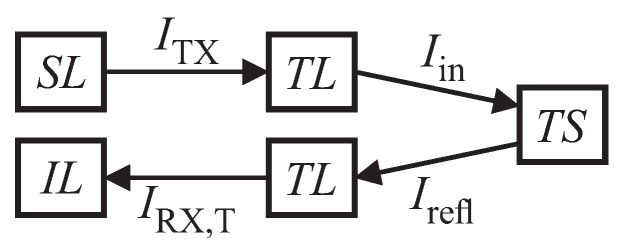
Acoustic intensities along transmission path; SL source level, $I_{\mathrm{TX}}$ transmitter intensity, TL transmission loss, $I_{\mathrm{in}}$ target input intensity, TS target strength, $I_{\mathrm{refl}}$ reflected target intensity, $I_{\mathrm{RX},T}$ input intensity from target and IL input level.

Figure 6 shows the relations for acoustic intensity levels along an echo’s transmission path, which can be put as follows (in analogy to the sonar equation from [43]):

(5) $$\begin{array}{cc} IL & =SL+TS-2TL\;\;\;\mathrm{in}\;\mathrm{dB}. \end{array}$$

Here, IL is the input level at the receiver, SL is the source level at the transmitter, TS is the target strength and TL is the transmission loss, respectively. For our calculations, the underlying assumption is that TL is only caused by geometric spreading. Compensation of frequency-dependent effects is left to the ANNs. The echo $p_{\mathrm{RX},T}\left(t\right)$ from the target can consist of multiple reflections *i* at times $t_{i}$ with different magnitudes $a_{i}$

(6) $$\begin{array}{cc} p_{\mathrm{RX},T} & =\sum_{i=1}^{N}a_{i}\;p_{\mathrm{TX}}\left(t-t_{i}\right)\;\;\;. \end{array}$$

The total target strength $TS_{1}$ for a target with a single echo, such as a disc or a cylinder, is used for *TStot* and consists of a variable part $\Delta TS_{1}$ as well as a constant part $TS_{1,\mathrm{const}}$

(7) $$\begin{array}{c} TS_{1}=2\;\left[\Delta TS_{1}+TS_{1,\mathrm{const}}\right]\;\;\;, \end{array}$$

with (see also Supplementary Materials)

(8) $$\begin{array}{cc} \Delta TS_{1} & =10\;\log_{10}\left(\frac{r_{\mathrm{yx}}^{T}\left(t_{T}\right)}{r_{0}}\right)+20\;\log_{10}\left(\frac{t_{T}}{t_{0}}\right) \end{array}$$

(9) $$\begin{array}{cc} \mathrm{and}\;\;\;TS_{1,\mathrm{const}} & =20\;\log_{10}\left(\frac{c\;t_{0}}{2\;\cdot \;1m}\right)-10\;\log_{10}\left(\frac{r_{\mathrm{xx},\mathrm{TX}}^{T}\left(0\right)}{r_{0}}\right)\;\;\;, \end{array}$$

where $t_{0}$ is an arbitrary time constant, $t_{T}$ is the target’s main peak in the pulse-compressed echo, $r_{\mathrm{yx}}^{T}\left(t_{T}\right)$ is the pulse-compressed echo’s value at $t_{T}$, $r_{0}$ can be chosen as an arbitrary constant, *c* is the speed of sound in air and $r_{\mathrm{xx},\mathrm{TX}}^{T}\left(0\right)$ is the acoustic excitation signal’s auto-correlation function value at time 0, respectively. For target echoes, $r_{t}$ is used for $r_{\mathrm{yx}}^{T}\left(t_{T}\right)$ and for non-target echoes, $r_{\mathrm{nt}}$ is used (compare Figure 5). We suppose that the impinging echo waves’ surface curvature across the microphone surface is negligible due to the small membrane area and the wave’s geometric spread over a large propagation distance in comparison, which leads to a plane wave assumption. Hence, intensity estimates are deduced from pressure measurements since sound pressure level and acoustic intensity level can be assumed to be equal for plane waves. This simplification suffices in our case as we are mainly interested in an estimate of target strength instead of a highly precise measurement. Compensation of possible variations due to the simplifications is left to the ANNs.

Apart from the total target strength $TS_{1}$, also the spectral target strength $\tilde{TS}\left(f\right)$ contains significant information for a target, especially for ones with multiple echoes such as a hollow hemisphere. $\tilde{TS}\left(f\right)$ depends on the frequency *f* and consists of a variable part $\Delta \tilde{TS}\left(f\right)$ as well as a constant part ${\tilde{TS}}_{\mathrm{const}}\left(f\right)$

(10) $$\begin{array}{cc} \tilde{TS}\left(f\right) & =2\;\left[\Delta \tilde{TS}\left(f\right)+{\tilde{TS}}_{\mathrm{const}}\left(f\right)\right]\;\;\;, \end{array}$$

(11) $$\begin{array}{cc} \mathrm{with}\;\;\;\Delta \tilde{TS}\left(f\right) & =10\;\log_{10}\left(\frac{S_{\mathrm{yx}}^{T}\left(f\right)}{S_{0}}\right)+20\;\log_{10}\left(\frac{t_{T}}{t_{0}}\right) \end{array}$$

(12) $$\begin{array}{cc} \mathrm{and}\;\;\;{\tilde{TS}}_{\mathrm{const}}\left(f\right) & =20\;\log_{10}\left(\frac{c\;t_{0}}{2\;\cdot \;1m}\right)-10\;\log_{10}\left(\frac{S_{\mathrm{xx},\mathrm{TX}}^{T}\left(f\right)}{S_{0}}\right)\;\;\;, \end{array}$$

where $S_{\mathrm{yx}}^{T}\left(f\right)$ denotes the cross-power-spectral density of the echo signal with the excitation signal, which can be obtained from $r_{\mathrm{yx}}^{T}\left(t\right)$ by Fourier transform. $S_{0}$ can be chosen arbitrarily and $S_{\mathrm{xx},\mathrm{TX}}^{T}\left(f\right)$ is the excitation signal’s auto-power-spectral density, which can be obtained from $r_{\mathrm{xx},\mathrm{TX}}^{T}\left(t\right)$ by Fourier transform. The derivation of the relations can be found in the Supplementary Materials.

We decided to represent the signal by an STFT for *TSspect* due to the same reasons as for the raw signal. The window size was chosen to be close to the excitation chirp duration (see [21] for considerations regarding STFT window size selection). Consequently, a window size of 1024 samples and an overlap of 50 are set for the STFT.

It is apparent from the TS equations that arbitrary excitation signals can be selected for target insonification. Therefore, use of rectified signals is possible and harmonic distortion is not a problem. We also performed tests with electrical sine as well as rectified chirp excitation and no difference in ANN performance results was detectable. Thus, less complex as well as smaller, lighter and cheaper amplifiers can be built for the ultrasonic speakers in comparison to amplifiers for sine signal excitation [44]. This is especially important for mobile robotic applications.

## 3. Results and Discussion

**Figure 7:**
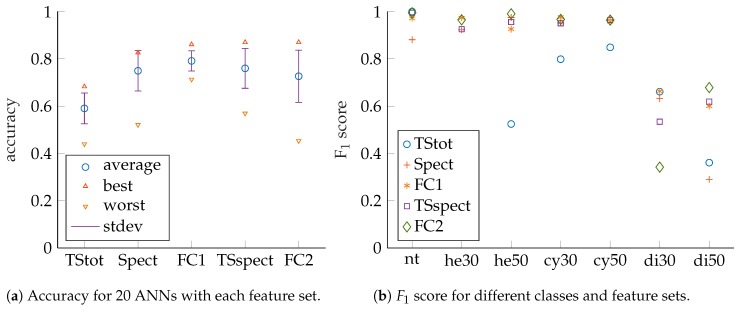
Performance measures for wideband excitation (150 kHz to 20 kHz). Compare also Table A1 and Table A2 in the Appendix A.

For each feature set, 20 ANNs were trained and evaluated by their accuracy as depicted in Figure 7a. The best as well as the worst ANN’s accuracy are shown for each feature set. In addition, mean accuracy as well as standard deviation are given and provide information regarding the number of necessary ANN training runs to find a well-performing solution. It can be seen that the best performing networks are found for *Spect*, *FC1*, *TSspect* and *FC2*. For those, best accuracies are close together. It can be concluded from Figure 7a that adding *TStot* to *Spect* (*FC1*) leads to noticeable improvement. We assume the ANNs do not need to learn how to devise *TStot* from spectral data by themselves in this case. Adding *TStot* to *TSspect* (*FC2*) leads to almost no noticeable improvement. Supposedly, this is the case since *TStot* will be easy to derive for an ANN if *TSspect* is given. If only *TStot* is used, it can be noticed that performance will not be as high as for the STFT-based features but also less effort for computation is necessary, as will be discussed later in this section. Consequently, *TStot* can be suitable for systems with limited resources, for which a tradeoff between classification accuracy and computational effort must be made.

**Figure 8:**
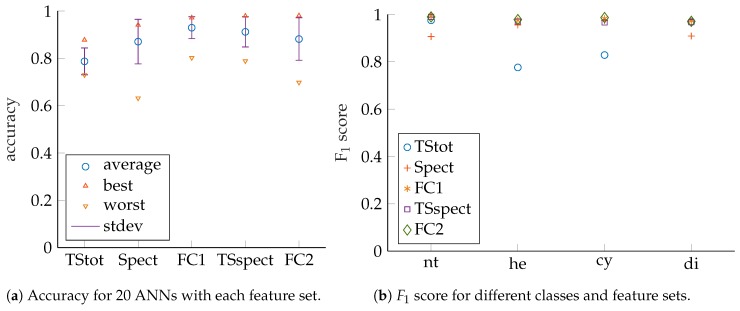
Performance measures for wideband excitation (150 kHz to 20kHz); grouped by shapes. Compare also Table A3 and Table A4 in the Appendix A.

We also looked at ANN performance for different classes with respect to the feature sets as indicated by the $F_{1}$ scores in Figure 7b. In this article, ANN accuracies and $F_{1}$ scores are presented, but for article preparation also confusion matrices as well as precision and recall values were additionally checked. The *x*-axis labels correspond to the following classes: *nt* no target, *he30* hemisphere 30 mm radius, *he50* hemisphere 50 mm radius, *cy30* cylinder 30 mm radius, *cy50* cylinder 50 mm radius, *di30* disc 30 mm radius, *di50* disc 50 mm radius, respectively. We compared the results of the networks which show the best accuracy for their feature sets, since those are the ones that would be selected for actual system deployment. It can be noticed that high scores are achieved for cylinders and hemispheres but that there is significant discrepancy for discs. Accordingly, disc size discrimination is the main challenge here and the target classes are thus regrouped by shapes to check that assumption, as shown in Figure 8a,b. So, only shape classification is performed and no size misclassifications are considered. It can be observed that significantly higher performance is achieved in this case, especially for disc-shaped targets. We suspect the main reason for difficulties with disc size discrimination is that small changes in insonification angle lead to drastic changes in echo magnitude. This can be deduced from the discs’ acoustic fingerprints for which there is only a very narrow angle that shows significant TS with a steep fall of TS with increasing angles (see Figure 3 for comparison).

**Figure 9:**
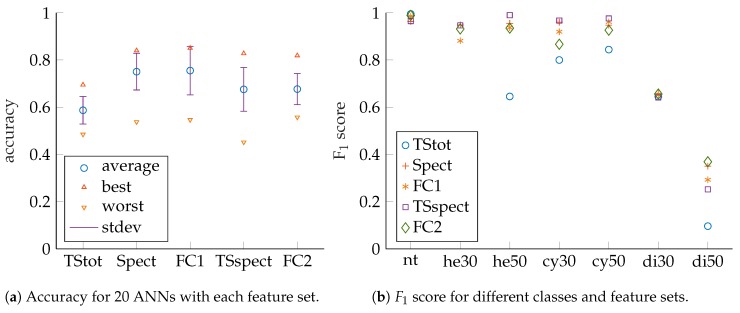
Performance measures for narrowband excitation (52 kHz to 48 kHz). Compare also Table A5 and Table A6 in the Appendix A.

**Figure 10:**
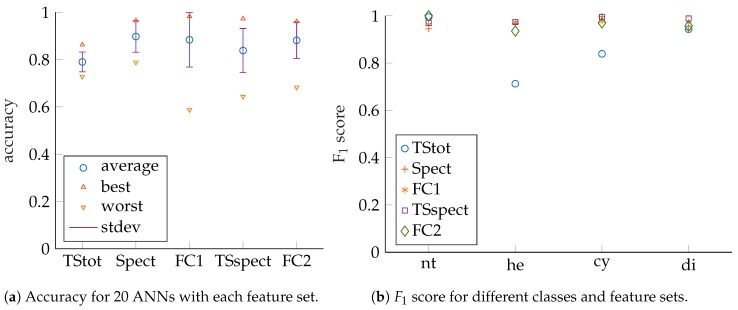
Performance measures for narrowband excitation (52 kHz to 48 kHz); grouped by shapes. Compare also Table A7 and Table A8 in the Appendix A.

Results for narrowband excitation are presented in Figure 9 and Figure 10. It can be seen that, for the given setup and task, the best results are close to the ones for wideband excitation. Accuracy values as well as $F_{1}$ scores are slightly lower for classification with respect to target size in comparison to the wideband case. Hence, additional information regarding target size will most likely be contained in wideband echo signals, which should consequently be used if targets of the same shape but differing sizes shall be set up and correctly identified in a robot environment. The targets that are approached by bats, such as small insects and plant pitchers as well as blossoms, are more diverse and show smaller geometric details, such as body and limb structure as well as leave shape, which lead to the necessity of wideband signals with narrow cross-correlation functions for proper resolution and subsequent classification by bats [12,13,14,15]. In contrast, the targets that are used for our application do not possess such small variations in geometry as well as size and, thus, narrowband excitation seems to suffice, especially if only target shapes shall be identified. For narrowband excitation, more training runs may be necessary to find best solutions for some features due to lower mean values. In addition, *TSspect* and *FC2* show reduced performance values in comparison to wideband excitation. Presumably, this is due to wider peaks in the calculated cross-correlation functions, which are caused by smaller signal bandwidth. Consequently, there are less sharp and distinctive features in the pulse-compressed waveforms, which need to be extracted by ANNs. The good narrowband results motivate replacement of the electrostatic speaker by robust and cheap piezoelectric transducers, which ought to be part of future work [30]. Furthermore, the measurement microphone may be replaced by MEMS microphones, which are also much lower in price and more robust [45,46].

Calculation times for ANN training, execution, feature preprocessing as well as total execution are presented in Table 1 as benchmarks to give the reader an impression of computation cost. Values were obtained on a desktop computer with Intel Core i7-8700 CPU, 32 GB RAM, Ubuntu 18.04 LTS operating system, Matlab R2018b and no GPU acceleration available. Averaging was performed across all samples and ANNs. It can be noticed that TStot calculations require the least time and that spectrogram-based features’ computation times are noticeably longer, with negligible differences amongst them. We presume the time increase is primarily caused by STFT calculations and larger feature vectors. Another observation that can be made is that ANN training for narrowband excitation takes longer than for the wideband case. Presumably, since more relevant information for classification needs to be extracted from a smaller frequency range. The benchmarks also indicate that an implementation with continuous operation should be possible. This is the case since the recording time for echo signals ( 15 ms in this contribution) is larger than total calculation times (preprocessing and ANN execution, less than 10 ms) and can therefore be finished before the end of the recording of subsequent echo signals. Please note that the currently implemented code is neither optimized for execution speed nor for hardware utilization and, consequently, even better performance should be possible. For embedded implementation on a robot, elaborate hardware/software codesign with widespread design space exploration is supposed to be beneficial to achieve an efficient real-time-capable implementation [47,48]. For that purpose, different hardware architectures can be employed, such as microcontrollers, DSPs (digital signal processors), FPGAs (Field Programmable Gate Arrays) or complete SOCs (Systems On Chip) [49,50,51]. The main processing steps—cross-correlation, peak detection, STFT and ANN execution—do have a major impact on calculation cost but can be parallelized to a high degree and also various other techniques can be employed to achieve a suitable implementation with regard to application requirements on aforementioned hardware. Potential solutions can comprise pipelining, divide and conquer approaches, approximate computing, replacement of float operations by integer arithmetic where applicable, LUTs (Look-Up Tables) etc. Also, calculations of constant parts of TStot and TSspect can be omitted (Equations (8) and (10)) because only their variable parts are relevant for target differentiation.

Certain limitations of the research need to be accounted for, which are to be addressed in ongoing as well as future work. Classification was performed with a well-defined set of targets and non-target echoes that can be well-separated from the targets. It thus remains to be investigated how robustly the system will perform if noisy echoes from cluttered spaces are included, which may often be the case for applications outside the laboratory environment. Most likely, narrowband performance will degrade as less spectral information is available. Additionally, we suspect that *TSspect* and *FC2* will perform noticeably better than *Spect* and *FC1* as uncorrelated parts of the recorded signals are filtered out during preprocessing.

## Figures and Tables

**Table 1:** Execution time benchmarks for wideband as well as narrowband excitation.

	Wideband					Narrowband				
	TStot	Spect	FC1	TSspect	FC2	TStot	Spect	FC1	TSspect	FC2
ANN train.	4.4 s	7.9 s	12.6 s	11.3 s	13.5 s	4.9 s	11.8 s	12.7 s	13.3 s	14.0 s
Preproc.	1.4 ms	3.7 ms	3.7 ms	3.9 ms	3.9 ms	1.4 ms	3.7 ms	3.7 ms	3.9 ms	3.9 ms
ANN exec.	4.9 ms	5.7 ms	5.7 ms	5.6 ms	5.6 ms	4.8 ms	5.6 ms	5.6 ms	5.7 ms	5.7 ms
Total exec.	6.3 ms	9.4 ms	9.5 ms	9.5 ms	9.5 ms	6.2 ms	9.3 ms	9.4 ms	9.6 ms	9.6 ms

## Supplementary Materials

The following are available online at https://www.mdpi.com/1424-8220/19/5/1176/s1.

## Abbreviations

The following abbreviations are used in this manuscript:

ANN	Artificial Neural Network
ROI	Region Of Interest
STFT	Short Time Fourier Transform
TS	Target Strength
RMS	Root Mean Square

## Appendix A. Performance Results

The results from Figure 7, Figure 8, Figure 9 and Figure 10 are given as tables in order to give the reader the opportunity to better identify as well as comprehend relations and differences in the data.

**Table A1:** Accuracy for wideband excitation (150 kHz to 20 kHz). Compare also Figure 7a.

	TStot	Spect	FC1	TSspect	FC2
**mean**	0.590	0.749	0.791	0.760	0.727
**stdev**	0.065	0.086	0.043	0.084	0.110
**best**	0.683	0.826	0.861	0.870	0.870
**worst**	0.440	0.521	0.713	0.569	0.453

**Table A2:** Best ANNs’ $F_{1}$ score for wideband excitation (150 kHz to 20 kHz). Compare also Figure 7b.

	TStot	Spect	FC1	TSspect	FC2
**nt**	1.000	0.881	0.971	0.995	0.995
**he30**	0.189	0.923	0.974	0.925	0.965
**he50**	0.524	0.975	0.926	0.956	0.991
**cy30**	0.799	0.952	0.976	0.950	0.967
**cy50**	0.849	0.968	0.958	0.964	0.964
**di30**	0.661	0.632	0.663	0.534	0.342
**di50**	0.361	0.290	0.600	0.619	0.679

**Table A3:** Accuracy for wideband excitation (150 kHz to 20 kHz); grouped by shapes. Compare also Figure 8a.

	TStot	Spect	FC1	TSspect	FC2
**mean**	0.787	0.870	0.929	0.912	0.881
**stdev**	0.057	0.094	0.046	0.064	0.090
**best**	0.878	0.941	0.971	0.979	0.980
**worst**	0.730	0.631	0.802	0.789	0.698

**Table A4:** Best ANNs’ $F_{1}$ score for wideband excitation (150 kHz to 20 kHz); grouped by shapes. Compare also Figure 8b.

	TStot	Spect	FC1	TSspect	FC2
**nt**	0.975	0.907	0.993	0.990	0.990
**he**	0.776	0.956	0.975	0.970	0.978
**cy**	0.828	0.974	0.983	0.966	0.987
**di**	0.968	0.909	0.972	0.968	0.971

**Table A5:** Accuracy for narrowband excitation (52 kHz to 48 kHz). Compare also Figure 9a.

	TStot	Spect	FC1	TSspect	FC2
**mean**	0.587	0.751	0.755	0.676	0.677
**stdev**	0.058	0.078	0.103	0.093	0.066
**best**	0.695	0.840	0.850	0.828	0.819
**worst**	0.485	0.538	0.547	0.452	0.557

**Table A6:** Best ANNs’ $F_{1}$ score for wideband excitation (52 kHz to 48 kHz). Compare also Figure 9b.

	TStot	Spect	FC1	TSspect	FC2
**nt**	0.995	0.963	0.9859	0.9648	0.989
**he30**	0.330	0.949	0.8813	0.9468	0.932
**he50**	0.646	0.955	0.9371	0.9898	0.935
**cy30**	0.800	0.960	0.9197	0.9670	0.867
**cy50**	0.844	0.947	0.9613	0.9760	0.926
**di30**	0.645	0.658	0.6525	0.6413	0.656
**di50**	0.096	0.350	0.2926	0.2522	0.369

**Table A7:** Accuracy for narrowband excitation (52 kHz to 48 kHz); grouped by shapes. Compare also Figure 10a.

	TStot	Spect	FC1	TSspect	FC2
**mean**	0.790	0.898	0.884	0.838	0.882
**stdev**	0.042	0.066	0.115	0.093	0.077
**best**	0.863	0.965	0.983	0.973	0.961
**worst**	0.728	0.789	0.588	0.644	0.683

**Table A8:** Best ANNs’ $F_{1}$ score for wideband excitation (52 kHz to 48 kHz); grouped by shapes. Compare also Figure 10b.

	TStot	Spect	FC1	TSspect	FC2
**nt**	0.995	0.945	0.963	0.970	1.000
**he**	0.713	0.967	0.973	0.974	0.936
**cy**	0.839	0.997	0.978	0.995	0.970
**di**	0.944	0.941	0.972	0.989	0.957

## References

[1] Kleeman L., Kuc R., Siciliano B., Khatib O. (2016). Sonar Sensing. Springer Handbook of Robotics.

[2] Steckel J., Peremans H. (2013). BatSLAM: Simultaneous localization and mapping using biomimetic sonar. PLoS ONE.

[3] Everett H.R. (1995). Sensors for Mobile Robots: Theory and Application/H.R. Everett.

[4] Przybyla R.J., Tang H.Y., Guedes A., Shelton S.E., Horsley D.A., Boser B.E. (2015). 3D Ultrasonic Rangefinder on a Chip. IEEE J. Solid-State Circuits.

[5] Vanderelst D., Steckel J., Boen A., Peremans H., Holderied M.W. (2016). Place recognition using batlike sonar. eLife.

[6] Thrun S., Burgard W., Fox D. (2006). Probabilistic Robotics.

[7] Rashidi P., Mihailidis A. (2013). A Survey on Ambient-Assisted Living Tools for Older Adults. IEEE J. Biomed. Health Inform..

[8] Dahl T., Boulos M. (2014). Robots in Health and Social Care: A Complementary Technology to Home Care and Telehealthcare?. Robotics.

[9] Hameed I.A. (2014). Intelligent Coverage Path Planning for Agricultural Robots and Autonomous Machines on Three-Dimensional Terrain. J. Intell. Robot. Syst..

[10] Roldán J.J., Garcia-Aunon P., Garzón M., de León J., Del Cerro J., Barrientos A. (2016). Heterogeneous Multi-Robot System for Mapping Environmental Variables of Greenhouses. Sensors.

[11] Bac C.W., van Henten E.J., Hemming J., Edan Y. (2014). Harvesting Robots for High-value Crops: State-of-the-art Review and Challenges Ahead. J. Field Robot..

[12] Siemers B.M., Schnitzler H.U. (2004). Echolocation signals reflect niche differentiation in five sympatric congeneric bat species. Nature.

[13] von Helversen D. (2004). Object classification by echolocation in nectar feeding bats: Size-independent generalization of shape. J. Comp. Physiol. A Neuroethol. Sens. Neural Behav. Physiol..

[14] Yovel Y., Franz M.O., Stilz P., Schnitzler H.U. (2011). Complex echo classification by echo-locating bats: A review. J. Comp. Physiol. A Neuroethol. Sens. Neural Behav. Physiol..

[15] Simon R., Holderied M.W., von Helversen O. (2006). Size discrimination of hollow hemispheres by echolocation in a nectar feeding bat. J. Exp. Biol..

[16] Goodfellow I., Bengio Y., Courville A. (2016). Deep Learning.

[17] Bishop C.M. (2006). Pattern Recognition and Machine Learning.

[18] Haykin S.S. (2009). Neural Networks and Learning Machines.

[19] Barshan B., Ayrulu B., Utete S.W. (2000). Neural Network-Based Target Differentiation Using Sonar for Robotics Applications. IEEE Trans. Robot. Autom..

[20] Ayrulu B., Barshan B. (2001). Neural networks for improved target differentiation and localization with sonar. Neural Netw..

[21] Dmitrieva M., Valdenegro-Toro M., Brown K., Heald G., Lane D., Ueda N. (2017). Object classification with convolution neural network based on the time-frequency representation of their echo. Proceedings of the 2017 IEEE 27th International Workshop on Machine Learning for Signal Processing (MLSP).

[22] Eliakim I., Cohen Z., Kosa G., Yovel Y. (2018). A fully autonomous terrestrial bat-like acoustic robot. PLoS Comput. Biol..

[23] Kroh P.K., Simon R., Rupitsch S.J. (2018). Classification of Sonar Targets in Air—A Neural Network Approach. Proceedings.

[24] Steckel J., Boen A., Peremans H. (2013). Broadband 3-D Sonar System Using a Sparse Array for Indoor Navigation. IEEE Trans. Robot..

[25] Vossiek M., Mágori V., Ermert H. (1994). An ultrasonic multielement sensor system for position invariant object identification. Proceedings of the IEEE Ultrasonics Symposium ULTSYM-94.

[26] Pullano S.A., Fiorillo A.S., La Gatta A., Lamonaca F., Carni D.L. (2016). Comprehensive system for the evaluation of the attention level of a driver. Proceedings of the 2016 IEEE International Symposium on Medical Measurements and Applications (MeMeA).

[27] Pullano S.A., Fiorillo A.S., Vanello N., Landini L. (2016). Obstacle detection system based on low quality factor ultrasonic transducers for medical devices. Proceedings of the 2016 IEEE International Symposium on Medical Measurements and Applications (MeMeA).

[28] Rupitsch S., Lerch R., Strobel J., Streicher A. (2011). Ultrasound transducers based on ferroelectret materials. IEEE Trans. Dielectr. Electr. Insul..

[29] Streicher A., Kaltenbacher M., Lerch R., Peremans H. (2005). Broadband EMFi ultrasonic transducer for bat research. Proceedings of the 2005 IEEE Ultrasonics Symposium.

[30] Rupitsch S.J. (2019). Piezoelectric Sensors and Actuators: Fundamentals and Applications.

[31] Dougherty G. (2013). Pattern Recognition and Classification.

[32] Metz C.E. (1978). Basic principles of ROC analysis. Semin. Nucl. Med..

[33] Fawcett T. (2006). An introduction to ROC analysis. Pattern Recognit. Lett..

[34] Skolnik M.I. (2008). Radar Handbook.

[35] Abraham D.A. (2017). Signal Processing. Applied Underwater Acoustics.

[36] Marage J.P., Mori Y. (2010). Sonar and Underwater Acoustics.

[37] Kiefer D.A., Fink M., Rupitsch S.J. (2017). Simultaneous Ultrasonic Measurement of Thickness and Speed of Sound in Elastic Plates Using Coded Excitation Signals. IEEE Trans. Ultrason. Ferroelectr. Freq. Control.

[38] Afouras T., Chung J.S., Senior A., Vinyals O., Zisserman A. (2018). Deep Audio-visual Speech Recognition. IEEE Trans. Pattern Anal. Mach. Intell..

[39] Connolly J.H., Edmonds E.A., Guzy J.J., Johnson S.R., Woodcock A. (1986). Automatic speech recognition based on spectrogram reading. Int. J. Man-Mach. Stud..

[40] Ganapathy S. (2017). Multivariate Autoregressive Spectrogram Modeling for Noisy Speech Recognition. IEEE Signal Process. Lett..

[41] Gemmeke J.F., Virtanen T., Hurmalainen A. (2011). Exemplar-Based Sparse Representations for Noise Robust Automatic Speech Recognition. IEEE Trans. Audio Speech Lang. Process..

[42] Zue V., Lamel L. (1986). An expert spectrogram reader: A knowledge-based approach to speech recognition. Proceedings of the CASSP ’86, IEEE International Conference on Acoustics, Speech, and Signal Processing.

[43] Hodges R.P. (2010). Underwater Acoustics: Analysis, Design, and Performance of Sonar.

[44] Pollakowski M., Ermert H. (1994). Chirp signal matching and signal power optimization in pulse-echo mode ultrasonic nondestructive testing. IEEE Trans. Ultrason. Ferroelectr. Freq. Control.

[45] Dokmanic I., Tashev I., Liu X. (2014). Hardware and algorithms for ultrasonic depth imaging. Proceedings of the 2014 IEEE 17th International Conference on Computational Science and Engineering (CSE).

[46] Das A., Tashev I., Mohammed S. (2017). Ultrasound based gesture recognition. Proceedings of the ICASSP 2017.

[47] Teich J. (2012). Hardware/Software Codesign: The Past, the Present, and Predicting the Future. Proc. IEEE.

[48] Hennessy J.L., Patterson D.A. (2012). Computer Architecture: A Quantitative Approach.

[49] Motamedi M., Gysel P., Akella V., Ghiasi S. (2016). Design space exploration of FPGA-based Deep Convolutional Neural Networks. Proceedings of the 2016 21st Asia and South Pacific Design Automation Conference (ASP-DAC).

[50] Qiu J., Song S., Wang Y., Yang H., Wang J., Yao S., Guo K., Li B., Zhou E., Yu J., Chen D., Greene J. (2016). Going Deeper with Embedded FPGA Platform for Convolutional Neural Network. Proceedings of the 2016 ACM/SIGDA International Symposium on Field-Programmable Gate Arrays—FPGA ’16.

[51] Hochradel K., Hohler T., Becher A., Wildermann S., Sutor A. (2018). Development of a multisensor array for localizing bats in space. J. Phys. Conf. Ser..

