# Supplementary material for: Classification of Sonar Targets in Air: A Neural Network Approach
Source: Sensors (Basel). 2019 Mar 7;19(5):1176. doi: 10.3390/s19051176 (PMC6427766; doi:10.3390/s19051176)
Supplement: Supplementary file 1 [file sensors-19-01176-s001.pdf]

Supplementary Materials: Classification of Sonar Targets in Air – A Neural Network Approach

Patrick K. Kroh, Ralph Simon and Stefan J. Rupitsch

1. Target Strength Calculations

1.1. Total Target Strength

Mathematical derivations are shown here for the target strength estimates in the manuscript.

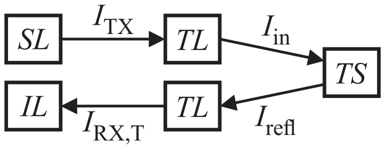

Figure S1. Acoustic intensities along transmission path; SL source level, I_{TX} transmitter intensity, TL transmission loss, I_{in} target input intensity, TS target strength, I_{refl} reflected target intensity, $I_{RX,T}$ input intensity from target and IL input level.

In analogy to the sonar equation as well as from Figure S1, sonar signal levels are given as

$$IL = SL + TS - 2TL \quad \text{in dB} \quad , \tag{1}$$

$$TS = 10 \log_{10}\left(\frac{I_{refl}}{I_{in}}\right) \quad , \tag{2}$$

$$IL = 10 \log_{10}\left(\frac{I_{RX,T}}{I_0}\right) \quad , \tag{3}$$

$$SL = 10 \log_{10}\left(\frac{I_{TX}}{I_0}\right) \tag{4}$$

$$\text{and} \quad TL = 10 \log_{10}\left(\frac{I_{in}}{I_{TX}}\right) = 10 \log_{10}\left(\frac{I_{RX}}{I_{refl}}\right) \quad , \tag{5}$$

with
IL: input level at receiver,
SL: source level at 1 m from transmitter,
TS: target strength,
TL: transmission loss,
I_{refl}: acoustic intensity after reflection from target at 1 m distance from target, $[I_{refl}] = 1\,\mathrm{W\,m^{-2}}$,
I_{in}: acoustic intensity at target position, $[I_{in}] = 1\,\mathrm{W\,m^{-2}}$,
$I_{RX,T}$: acoustic intensity at receiver due to target echo, $[I_{RX}] = 1\,\mathrm{W\,m^{-2}}$,
I_0: reference acoustic intensity, $I_0 = 1 \times 10^{-12}\,\mathrm{W\,m^{-2}}$,
I_{TX}: acoustic intensity at 1 m from transmitter, $[I_{TX}] = 1\,\mathrm{W\,m^{-2}}$,
All acoustic intensities are RMS values.

Since only geometric spreading is considered for TL, there is no dependency of frequency

$$TL = 20 \log_{10}\left(\frac{R}{1\,\mathrm{m}}\right) = 20 \log_{10}\left(\frac{t_T\, c}{2 \cdot 1\,\mathrm{m}}\right) \quad , \tag{6}$$

with
R: sound propagation distance (between speaker/microphone and target),
t_T: time delay for echo from target in s,

c: speed of sound, $343\,\mathrm{m\,s^{-1}}$ in air.

The relation between acoustic intensity and a pressure signal's auto-correlation function is based on a plane wave assumption (due to small echo wave surface curvature across microphone area) and is given as

$$I = \frac{1}{Z_0}\, r^{\mathrm{T}}_{\mathrm{aa}}(0) \quad , \tag{7}$$

with
I: acoustic intensity RMS in $\mathrm{W\,m^{-2}}$,
Z_0: characteristic specific acoustic impedance in $\mathrm{Pa\,s\,m^{-1}}$,
$r^{\mathrm{T}}_{\mathrm{aa}}(0)$: central peak of short time ACF of pressure signal in Pa^2.

We used the following definition for a short time XCF $r_{\mathrm{ab}}(\tau)$ of two arbitrary pressure signals p_{a} and p_{b}

$$r^{\mathrm{T}}_{\mathrm{ab}}(\tau) = \frac{1}{2\,T_{\mathrm{ref}}} \int_{-\infty}^{+\infty} p_{\mathrm{a}}(t+\tau)\, p_{\mathrm{b}}(t)\, \mathrm{d}t \quad , \tag{8}$$

with
T_{ref}: reference time, e.g., excitation signal duration, in s,
t, τ: time variables in s.

Based on correlation, factors from the sonar equation can be expressed as

$$TS = 10\,\log_{10}\left(\frac{r^{\mathrm{T}}_{\mathrm{xx,refl}}(0)}{r^{\mathrm{T}}_{\mathrm{xx,in}}(0)}\right) \quad , \tag{9}$$

$$IL = 10\,\log_{10}\left(\frac{r^{\mathrm{T}}_{\mathrm{xx,RX,T}}(0)}{r_0}\right) \tag{10}$$

$$\text{and} \quad SL = 10\,\log_{10}\left(\frac{r^{\mathrm{T}}_{\mathrm{xx,TX}}(0)}{r_0}\right) \quad , \tag{11}$$

with
$r^{\mathrm{T}}_{\mathrm{xx,refl}}(\tau)$: short time ACF of acoustic pressure $p_{\mathrm{refl}}(t)$ at 1 m distance from target, in Pa^2,
$r^{\mathrm{T}}_{\mathrm{xx,in}}(\tau)$: short time ACF of acoustic pressure $p_{\mathrm{in}}(t)$ at target position, in Pa^2,
$r^{\mathrm{T}}_{\mathrm{xx,RX,T}}(\tau)$: short time ACF of acoustic pressure $p_{\mathrm{RX,T}}(t)$ at receiver due to echo from target, in Pa^2,
r_0: reference value for correlation functions of acoustic pressure, $r_0 = I_0 Z_0 = p_0^2$, in Pa^2,
$r^{\mathrm{T}}_{\mathrm{xx,TX}}(\tau)$: short time ACF of acoustic pressure $p_{\mathrm{TX}}(t)$ at 1 m from transmitter, in Pa^2.

The pressure signal at the receiver is given as

$$p_{\mathrm{RX}}(t) = p_{\mathrm{RX,T}}(t) + p_{\mathrm{uncorr}}(t) \quad , \tag{12}$$

with
$p_{\mathrm{RX}}(t)$: acoustic pressure signal at the receiver, in Pa
$p_{\mathrm{RX,T}}(t)$: echo from target at the receiver, in Pa
$p_{\mathrm{uncorr}}(t)$: uncorrelated acoustic pressure, not from target echo, e.g., noise and other ultrasonic sound

sources, in Pa.

Multiple echoes i can occur at a target such as a hollow hemisphere:

$$p_{\mathrm{RX,t}} = \sum_{i=1}^{N} a_i \, p_{\mathrm{TX}}(t - t_i) \tag{13}$$

$$\text{with} \quad t_i = t_{\mathrm{T}} + \Delta t_i, \tag{14}$$

$$\Delta t_{i+1} > \Delta t_i \tag{15}$$

$$\text{and} \quad \Delta t_1 = 0, \tag{16}$$

with
i: echo number, starts at 1,
N: number of reflections,
a_i: relative magnitude of single echo,
t_i: propagation delay for echo i,
Δt_i: delay difference for echo i.

Cross-correlation between p_{RX} and p_{TX} leads to pulse-compression and thus removal of uncorrelated parts from the echo signal:

$$r_{\mathrm{yx}}^{\mathrm{T}}(\tau) = \sum_{i=1}^{N} a_i \, r_{\mathrm{xx,TX}}^{\mathrm{T}}(\tau - t_i) \tag{17}$$

with
$r_{\mathrm{yx}}^{\mathrm{T}}(t_{\mathrm{T}})$: pulse-compressed echo's value at t_{T}
(see Appendix 2.1 for detailed calculations).

A relation between $r_{\mathrm{xx,RX,T}}^{\mathrm{T}}(\tau)$ and $r_{\mathrm{yx}}^{\mathrm{T}}(\tau)$ can be derived, too:

$$r_{\mathrm{xx,RX,T}}^{\mathrm{T}}(\tau) = \sum_{i=1}^{N} a_i \, r_{\mathrm{yx}}^{\mathrm{T}}(\tau + t_i) \tag{18}$$

(see Appendix 2.2 for detailed calculations).

As a consequence, it can be inferred for a reflector with a single dominant echo (e.g., disc and cylinder) that

$$TS_1 = 20 \, \log_{10}\left(\frac{r_{\mathrm{yx}}^{\mathrm{T}}(t_T)}{r_{\mathrm{xx,TX}}^{\mathrm{T}}(0)}\right) + 40 \, \log_{10}\left(\frac{t_{\mathrm{T}} \, c}{2 \cdot 1\,\mathrm{m}}\right) \tag{19}$$

$$= 2\,(\Delta TS_1 + TS_{1,\mathrm{const}}) \tag{20}$$

with
TS_1: total target strength estimate
t_{T}: target's main peak delay in pulse-compressed echo
ΔTS_1: relative total target strength estimate,
$TS_{1,\mathrm{const}}$: constant part of total target strength estimate
with

$$\Delta TS_1 = 10\,\log_{10}\left(\frac{r^{\mathrm{T}}_{\mathrm{yx}}(t_{\mathrm{T}})}{r_0}\right) + 20\,\log_{10}\left(\frac{t_{\mathrm{T}}}{t_0}\right) \tag{21}$$

$$\text{and} \quad TS_{1,\mathrm{const}} = 20\,\log_{10}\left(\frac{c\,t_0}{2\cdot 1\,\mathrm{m}}\right) - 10\,\log_{10}\left(\frac{r^{\mathrm{T}}_{\mathrm{xx,TX}}(0)}{r_0}\right) \tag{22}$$

with
r_0: arbitrary constant in Pa^2,
t_0: arbitrary time constant in s.

1.2. Spectral Target Strength

Spectral target strength relations:

$$TS = 10\,\log_{10}\left(\frac{\int_{-\infty}^{+\infty} S^{\mathrm{T}}_{\mathrm{xx,refl}}(f)\,\mathrm{d}f}{\int_{-\infty}^{+\infty} S^{\mathrm{T}}_{\mathrm{xx,in}}(f)\,\mathrm{d}f}\right) = 10\,\log_{10}\left(\frac{\int_{-\infty}^{+\infty} S^{\mathrm{T}}_{\mathrm{xx,in}}(f)\,\widetilde{TS}_{lin}(f)\,\mathrm{d}f}{\int_{-\infty}^{+\infty} S^{\mathrm{T}}_{\mathrm{xx,in}}(f)\,\mathrm{d}f}\right) \quad , \tag{23}$$

$$\widetilde{TS}(f) = 10\,\log_{10}\left(\frac{S^{\mathrm{T}}_{\mathrm{xx,refl}}(f)}{S^{\mathrm{T}}_{\mathrm{xx,in}}(f)}\right) \quad , \tag{24}$$

$$IL = 10\log_{10}\left(\frac{\int_{-\infty}^{+\infty} S^{\mathrm{T}}_{\mathrm{xx,RX,T}}(f)\,\mathrm{d}f}{r_0}\right) \tag{25}$$

$$\text{and} \quad SL = 10\,\log_{10}\left(\frac{\int_{-\infty}^{+\infty} S^{\mathrm{T}}_{\mathrm{xx,TX}}(f)\,\mathrm{d}f}{r_0}\right) \tag{26}$$

with
$S^{\mathrm{T}}_{\mathrm{xx,refl}}(f)$: auto power spectral density for $p_{\mathrm{refl}}(t)$, in $\mathrm{Pa}^2\,\mathrm{Hz}^{-1}$,
$S^{\mathrm{T}}_{\mathrm{xx,in}}(f)$: auto power spectral density for $p_{\mathrm{in}}(t)$, in $\mathrm{Pa}^2\,\mathrm{Hz}^{-1}$,
$\widetilde{TS}(f)$: spectral target strength,
$S^{\mathrm{T}}_{\mathrm{xx,RX,T}}(f)$: auto power spectral density for $p_{\mathrm{RX,T}}(t)$, in $\mathrm{Pa}^2\,\mathrm{Hz}^{-1}$,
$S^{\mathrm{T}}_{\mathrm{xx,TX}}(f)$: auto power spectral density for $p_{\mathrm{TX}}(t)$, in $\mathrm{Pa}^2\,\mathrm{Hz}^{-1}$.

Relations between correlation functions and spectral properties are

$$S^{\mathrm{T}}_{\mathrm{ab}}(f) = R^{\mathrm{T}}_{\mathrm{ab}}(f) = \mathcal{F}\{r^{\mathrm{T}}_{\mathrm{ab}}(t)\} \tag{27}$$

$$\text{and} \quad r^{\mathrm{T}}_{\mathrm{ab}}(t) = \int_{-\infty}^{+\infty} S^{\mathrm{T}}_{\mathrm{ab}}(f)\,\mathrm{e}^{\mathrm{j}2\pi f t}\,\mathrm{d}f \tag{28}$$

with
$S^{\mathrm{T}}_{\mathrm{ab}}(f)$: cross power spectral density for two arbitrary signals $p_{\mathrm{a}}(t)$ and $p_{\mathrm{b}}(t)$, here in $\mathrm{Pa}^2\,\mathrm{Hz}^{-1}$,
$R^{\mathrm{T}}_{\mathrm{ab}}(f)$: fourier transform of cross correlation of $p_{\mathrm{a}}(t)$ and $p_{\mathrm{b}}(t)$, here in $\mathrm{Pa}^2\,\mathrm{s}$,
$\mathcal{F}\{\cdots\}$: fourier transform.

From equations 17 and 18 as well as 28, it follows that

$$S_{\mathrm{yx}}^{\mathrm{T}}(f) = S_{\mathrm{xx,TX}}^{\mathrm{T}}(f) \sum_{i=1}^{N} a_i \, \mathrm{e}^{-\mathrm{j}2\pi f t_i} \tag{29}$$

$$\text{and} \quad S_{\mathrm{xx,RX,T}}^{\mathrm{T}}(f) = S_{\mathrm{yx}}^{\mathrm{T}}(f) \sum_{i=1}^{N} a_i \, \mathrm{e}^{\mathrm{j}2\pi f t_i} \tag{30}$$

with
$S_{\mathrm{yx}}^{\mathrm{T}}(f)$: cross-power-spectral density of echo signal with the excitation signal.

We can write

$$10 \log_{10}\left(\frac{I_{\mathrm{in}}}{I_0}\right) = SL - TL \tag{31}$$

and thus

$$S_{\mathrm{xx,in}}^{\mathrm{T}}(f) = S_{\mathrm{xx,TX}}^{\mathrm{T}}(f) \, \frac{R^2}{1\,\mathrm{m}^2} \quad , \tag{32}$$

$$TS = 10 \log_{10}\left(\frac{\int_{-\infty}^{+\infty} S_{\mathrm{xx,TX}}^{\mathrm{T}}(f) \, \widetilde{TS}_{\mathrm{lin}}(f) \, \mathrm{d}f}{\int_{-\infty}^{+\infty} S_{\mathrm{xx,TX}}^{\mathrm{T}}(f) \, \mathrm{d}f}\right) \tag{33}$$

$$\text{and} \quad \widetilde{TS}(f) = 10 \log_{10}\left(\frac{S_{\mathrm{xx,RX,T}}^{\mathrm{T}}(f)}{S_{\mathrm{xx,TX}}^{\mathrm{T}}(f)}\right) + 2\,TL \quad , \tag{34}$$

(see Appendix 2.3 for detailed calculations)
so as a consequence, we obtain

$$\widetilde{TS}(f) = 20 \log_{10}\left(\frac{S_{\mathrm{yx}}^{\mathrm{T}}(f)}{S_{\mathrm{xx,TX}}^{\mathrm{T}}(f)}\right) + 2\,TL \tag{35}$$

$$\text{and} \quad \widetilde{TS}(f) = 2\,(\Delta\widetilde{TS}(f) + \widetilde{TS}_{\mathrm{const}}(f)) \tag{36}$$

with
$\Delta\widetilde{TS}$: relative spectral target strength estimate,
$\widetilde{TS}_{\mathrm{const}}$: constant part of spectral target strength,
which are calculated by

$$\Delta\widetilde{TS}(f) = 10 \log_{10}\left(\frac{S_{\mathrm{yx}}^{\mathrm{T}}(f)}{S_0}\right) + 20 \log_{10}\left(\frac{t_{\mathrm{T}}}{t_0}\right) \tag{37}$$

$$\text{and} \quad \widetilde{TS}_{\mathrm{const}}(f) = 20 \log_{10}\left(\frac{c\,t_0}{2 \cdot 1\,\mathrm{m}}\right) - 10 \log_{10}\left(\frac{S_{\mathrm{xx,TX}}^{\mathrm{T}}(f)}{S_0}\right) \tag{38}$$

with
S_0: power spectral density reference value, may be chosen arbitrarily, in $\mathrm{Pa}^2\,\mathrm{Hz}^{-1}$.

2. Detailed Proof of Relations Between Signal Correlation Functions

2.1. Proof for equation 17

The XCF of p_{RX} and p_{TX} can be calculated by

$$r^{\mathrm{T}}_{\mathrm{yx}}(\tau) = \frac{1}{2\,T_{\mathrm{ref}}} \int_{-\infty}^{+\infty} p_{\mathrm{RX}}(t+\tau)\, p_{\mathrm{TX}}(t)\,\mathrm{d}t \quad , \tag{39}$$

$$= \frac{1}{2\,T_{\mathrm{ref}}} \int_{-\infty}^{+\infty} \left[\sum_{i=1}^{N} a_i\, p_{\mathrm{TX}}(t-t_i+\tau) + p_{\mathrm{uncorr}}(t+\tau)\right] p_{\mathrm{TX}}(t)\,\mathrm{d}t \quad , \tag{40}$$

$$= \frac{1}{2\,T_{\mathrm{ref}}} \sum_{i=1}^{N} a_i \int_{-\infty}^{+\infty} p_{\mathrm{TX}}(t-t_i+\tau)\, p_{\mathrm{TX}}(t)\,\mathrm{d}t \quad , \tag{41}$$

$$= \sum_{i=1}^{N} a_i\, r^{\mathrm{T}}_{\mathrm{xx,TX}}(\tau - t_i) \quad . \tag{42}$$

p_{uncorr} is assumed to be orthogonal to p_{TX} and, therefore, does not have an influence on $r^{\mathrm{T}}_{\mathrm{yx}}(\tau)$.

2.2. Proof for equation 18

The relation between the target echo ACF $r^{\mathrm{T}}_{\mathrm{xx,RX,T}}(\tau)$ and $r^{\mathrm{T}}_{\mathrm{yx}}(\tau+t_i)$ is derived by

$$r^{\mathrm{T}}_{\mathrm{xx,RX,T}}(\tau) = \frac{1}{2\,T_{\mathrm{ref}}} \int_{-\infty}^{+\infty} p_{\mathrm{RX,T}}(t+\tau)\, p_{\mathrm{RX,T}}\,\mathrm{d}t \quad , \tag{43}$$

$$= \frac{1}{2\,T_{\mathrm{ref}}} \int_{-\infty}^{+\infty} \sum_{i=1}^{N} a_i\, p_{\mathrm{TX}}(t-t_i+\tau) \sum_{k=1}^{N} a_k\, p_{\mathrm{TX}}(t-t_k)\,\mathrm{d}t \quad , \tag{44}$$

$$= \sum_{i=1}^{N} a_i \sum_{k=1}^{N} a_k \frac{1}{2T_{\mathrm{ref}}} \int_{-\infty}^{+\infty} p_{\mathrm{TX}}(t-t_i+\tau)\, p_{\mathrm{TX}}(t-t_k)\,\mathrm{d}t \quad , \tag{45}$$

$$= \sum_{i=1}^{N} a_i \sum_{k=1}^{N} a_k \frac{1}{2T_{\mathrm{ref}}} \int_{-\infty}^{+\infty} p_{\mathrm{TX}}(t-t_i+t_k+\tau)\, p_{\mathrm{TX}}(t)\,\mathrm{d}t \quad , \tag{46}$$

$$= \sum_{i=1}^{N} a_i \sum_{k=1}^{N} a_k\, r^{\mathrm{T}}_{\mathrm{xx,TX}}(\tau - t_i + t_k) \quad , \tag{47}$$

$$= \sum_{i=1}^{N} a_i\, r^{\mathrm{T}}_{\mathrm{yx}}(\tau + t_i) \quad . \tag{48}$$

Equation 17 is used in the last step.

2.3. Proof for equation 32

From equation 31 follows

$$10\,\log_{10}\left(\frac{I_{\mathrm{in}}}{I_{\mathrm{TX}}}\right) = TL \tag{49}$$

$$\text{and} \quad 10\,\log_{10}\left(\frac{\int_{-\infty}^{+\infty} S^{\mathrm{T}}_{\mathrm{xx,in}}(f)\,\mathrm{d}t}{\int_{-\infty}^{+\infty} S^{\mathrm{T}}_{\mathrm{xx,TX}}(f)\,\mathrm{d}t}\right) = 10\,\log_{10}\left(\frac{R^2}{1\,\mathrm{m}^2}\right), \tag{50}$$

where TL does not depend on frequency and the relation must be true for any arbitrary $S^{\mathrm{T}}_{\mathrm{xx,in}}(f)$. Hence, the relation

$$10 \log_{10}\left(\frac{S^{\mathrm{T}}_{\mathrm{xx,in}}(f)}{S^{\mathrm{T}}_{\mathrm{xx,TX}}(f)}\right) = 10 \log_{10}\left(\frac{R^2}{1\,\mathrm{m}^2}\right) \tag{51}$$

must be true.

Abbreviations

The following abbreviations are used in this manuscript:

TS	Target Strength
RMS	Root Mean Square
ACF	Auto-Correlation Function
XCF	Cross-Correlation Function
